# Supplementary material for: A Novel Model for the RNase MRP-Induced Switch between the Formation of Different Forms of 5.8S rRNA
Source: Int J Mol Sci. 2021 Jun 22;22(13):6690. doi: 10.3390/ijms22136690 (PMC8268776; doi:10.3390/ijms22136690)
Supplement: Supplementary file 1 [file ijms-22-06690-s001.zip › ijms-1219611-supplementary.pdf]

Supplementary Materials

Table S1. Long 5.8S rRNA normalized to total 5.8S (long+short). Average of 25° and 37° samples

ITS1 mutant	*Δrpa12 Δ xrn*			YLL53 (*RRP2*)			YLL54 (*rrp2-2*)			Average of Δrpa12Δxrn1 and YLL53		
	Ave	STD	n	Ave	STD	n	Ave	STD	n	Ave	STD	n
wt	0,21	0,044	10	0,20	0,034	8	0,59	0,042	8	0,21	0,039	18
Δ13	0,19	0,015	2	0,21	0,045	2	0,76	0,034	2	0,20	0,030	4
Δ14	0,31	0,025	2	0,34	0,036	2	0,80	0,026	2	0,33	0,033	4
Δ15	0,26	0,026	2	0,40	0,015	4	0,60	0,093	6	0,34	0,085	6
Δ16	0,22	0,000	2	0,34	0,036	2	0,80	0,026	2	0,18	0,045	4
Δ17	0,61	0,002	2	0,34	0,036	2	0,80	0,026	2	0,75	0,157	4
Δ18	0,50	0,122	2	0,34	0,036	2	0,80	0,026	2	0,65	0,185	4
Δ19	0,56	0,030	2	0,64	--	1	0,81	0,044	2	0,61	0,050	3
Δ2	0,74	0,007	4	0,82	0,028	2	0,89	0,002	2	0,77	0,042	6

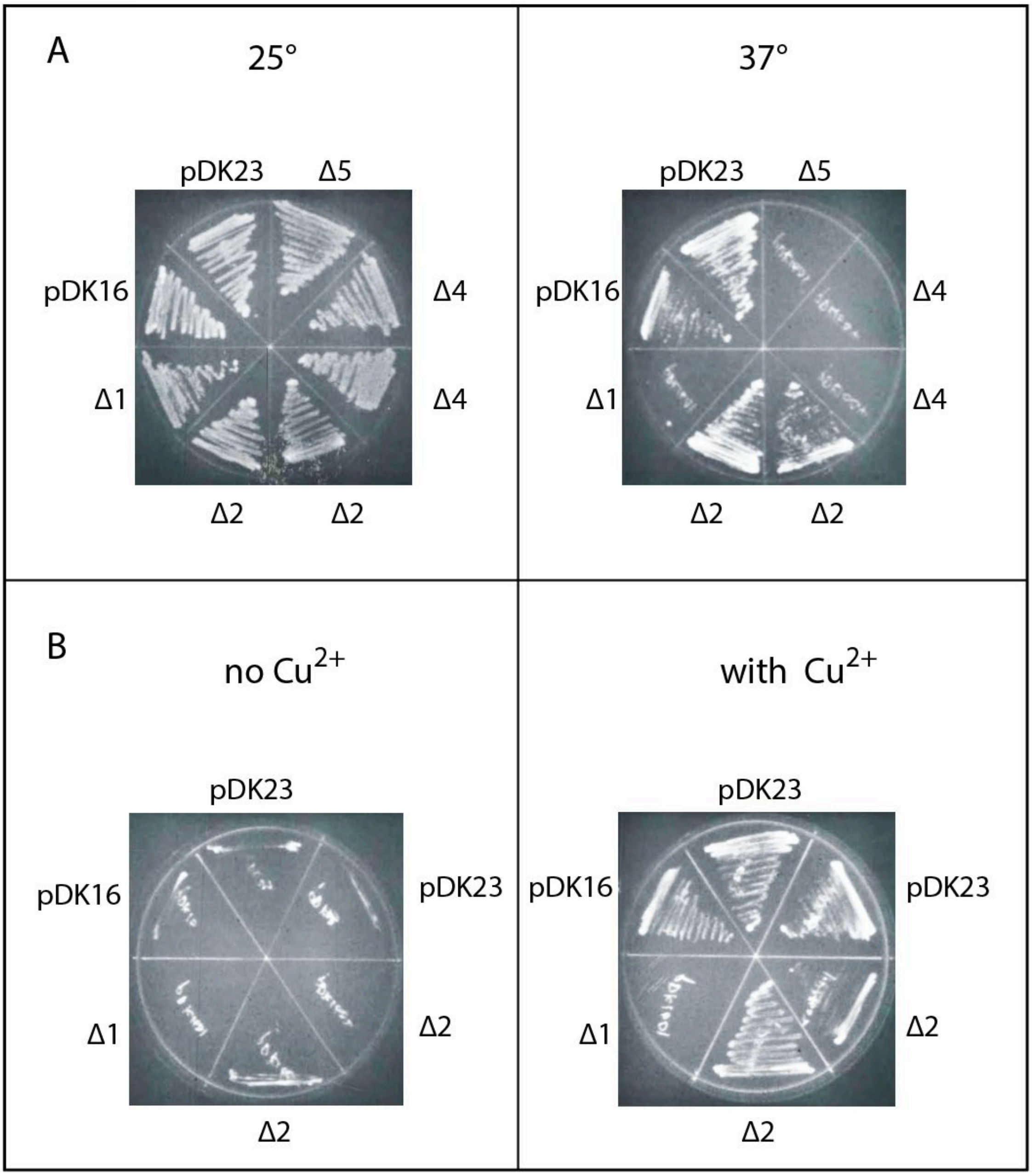

Figure S1. Test of ITS1 deletions for support of growth. pDK16 and derivatives with deletions inside ITS1 were transformed into *Δrpa135* and *Δrpa12* selecting for tryptophan prototrophy. (**A**) *Δrpa12* transformants were streaked on on synthetic glucose medium with Cu^{2+} and incubated at 25° and 37°. pDK16 and pDK16Δ2 are described in the text. pDK23 contains a 7 basepair deletion in the A3 site [35]. Δ1 is missing the entire 5.8S, ITS2 and the first 318 nucleotides of 25S. Δ5 is a combination of Δ2 and Δ4. (**B**) *Δrpa135* transformants were streaked on synthetic glucose plates (repressing expression of rRNA from the resident Pgal-rRNA plasmid) with and without Cu^{2+} and incubated at 30°.

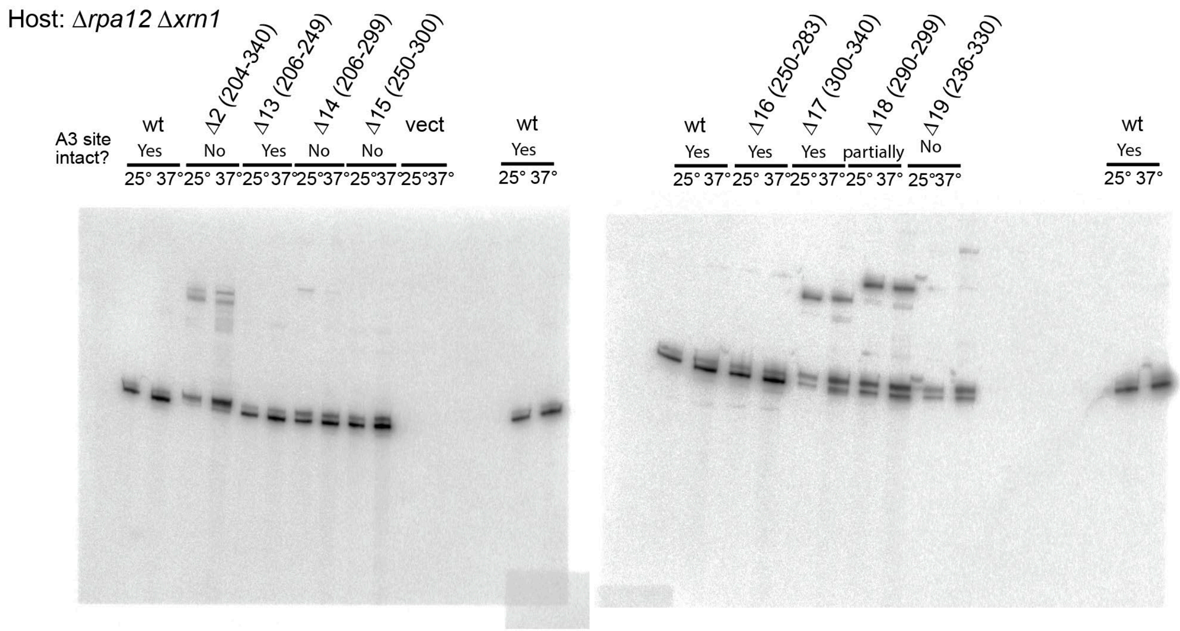

Figure S2. Uncropped autoradiograms used for Figure 3 top row.

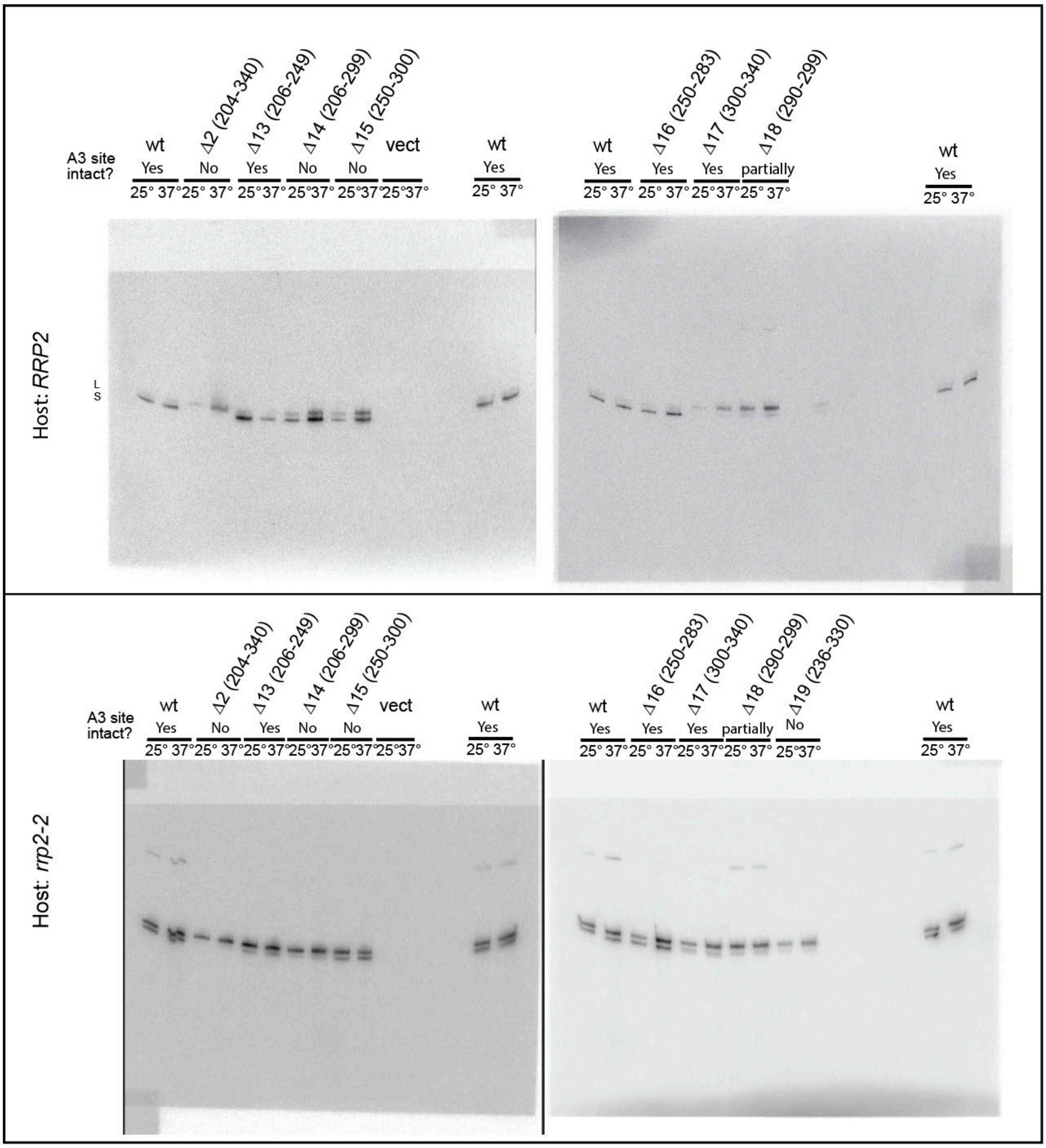

Figure S3. Uncropped autoradiograms used for Figure 3 middle and bottom rows.

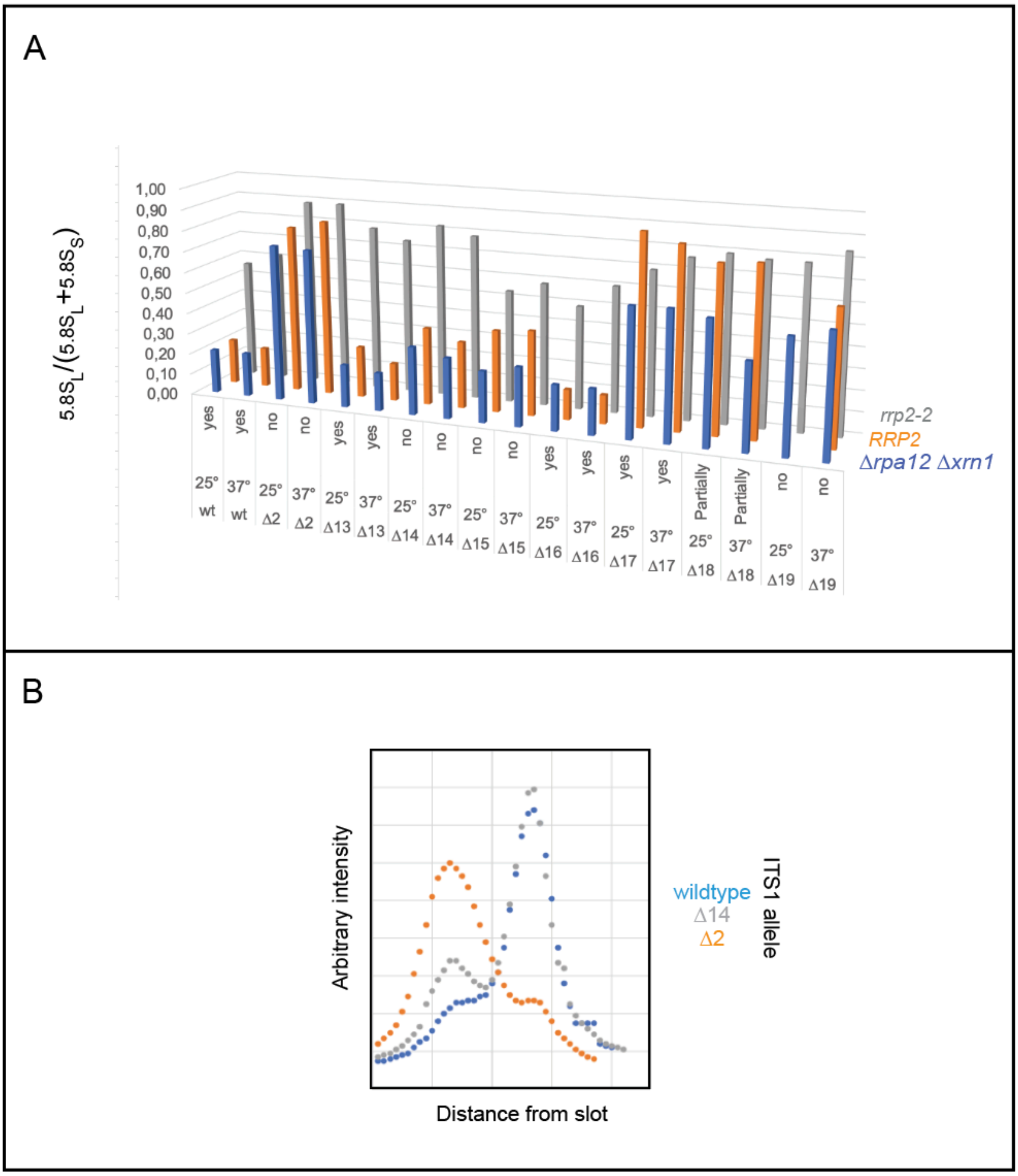

Figure S4. Quantification of long and short 5.8S rRNA in strains carrying pDK16 or its ITS1 deletion derivatives. Cultures of *Δrpa12 Δxrn1* and *RRP2* (YLL53) and its *rrp2-2* isogenic sibling (YLL54) harboring pDK16, or one of its ITS1 deletion derivatives, were grown at 25° and shifted to 37° for 6 hours. Total RNA was extracted from samples of the culture before and after the shift and used for acrylamide gel electrophoresis followed by northern blot analysis using O576 probe (Figure 3 in main text). The bands were quantified using Adobe Photoshop and the fraction of total 5.8S rRNA constituted by 5.8S$_L$ (L-fraction) was calculated for each sample. The results were graphed using Microsoft Excel (panel A). Examples of the resolution of lanes with RNA from wildtype (low 5.8S$_L$), Δ14 (medium 5.8S$_L$), and Δ2 in *Δrpa12 Δxrn1* are shown in panel B. Since there is little difference in the L-fraction for samples harvested before and after the temperature shift, the results from the 25° and 37° samples from a given condition were averaged and results are shown in Figure 5A.

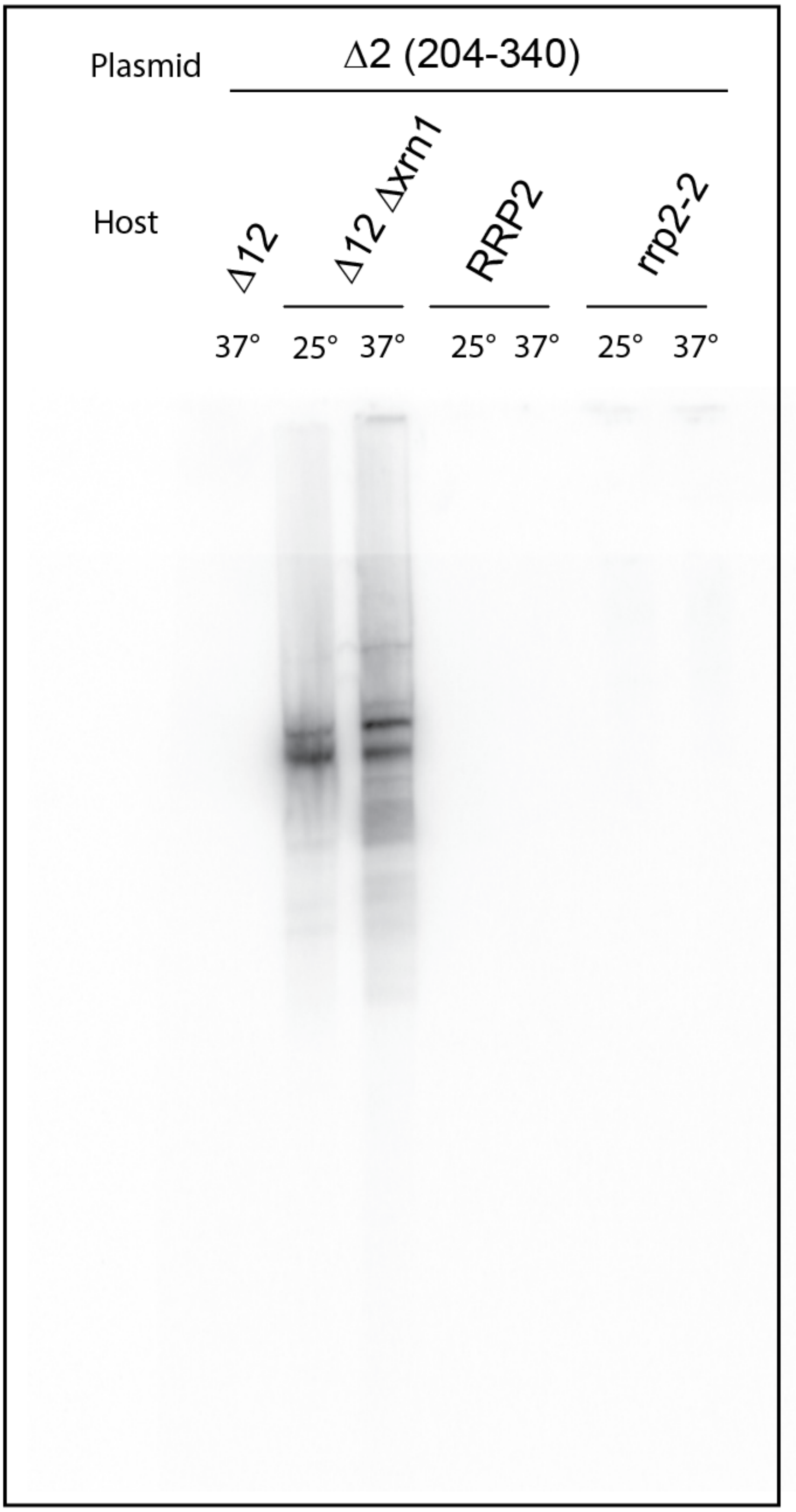

Figure S5. Uncropped northern blot of RNA from *Δrpa12*, *Δrpa12 Δxrn1*, *RRP2*, and *rrp2-2*, all carrying pDK16Δ2 probed with O552 spanning the Δ2 deletion and revealing RNA transcripts containing the Δ2 deletion (see text).

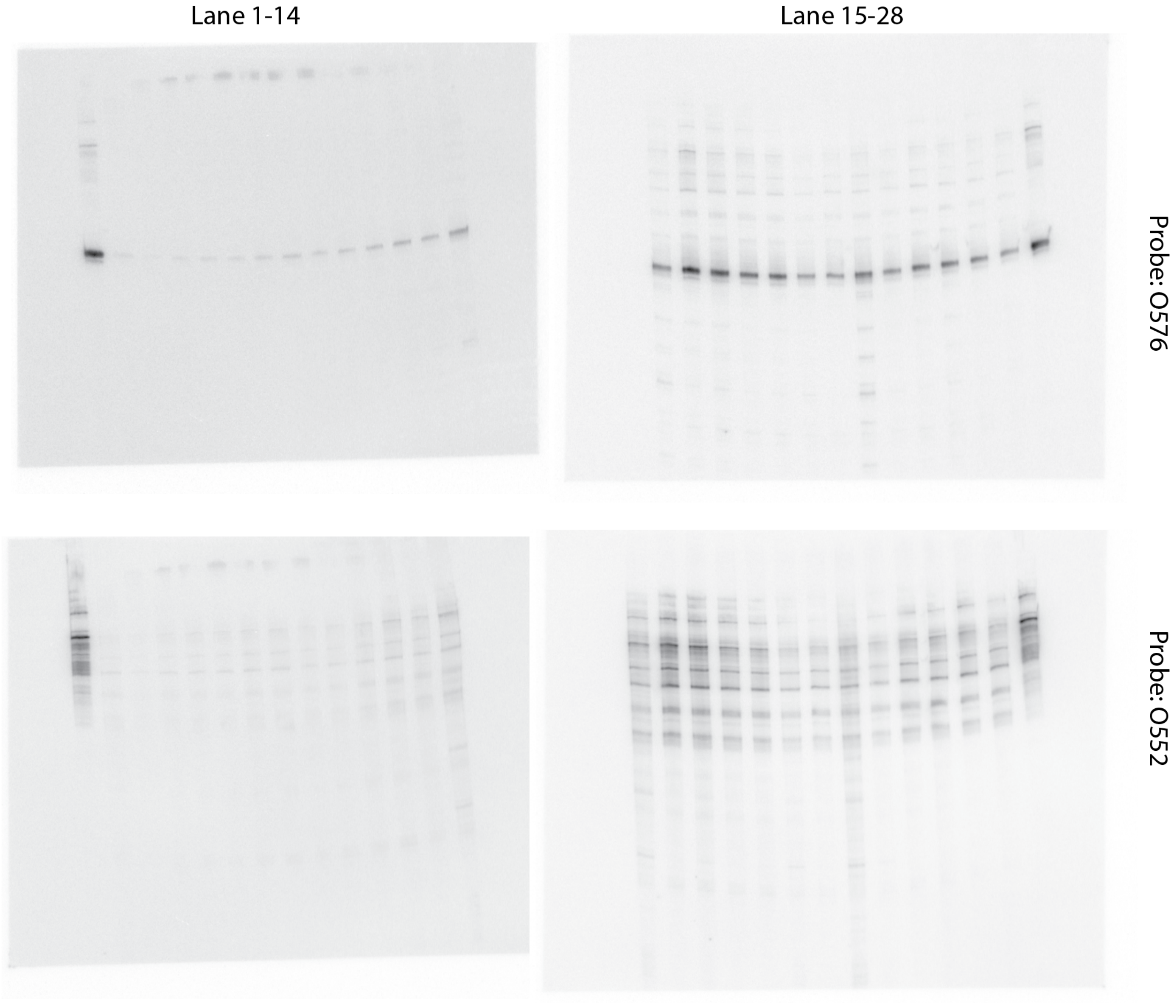

Figure S6. Uncropped northern blots used for Figure 8.
